# The Ultrasmall Biocompatible CuS@BSA Nanoparticle and Its Photothermal Effects

**DOI:** 10.3389/fphar.2019.00141

**Published:** 2019-02-26

**Authors:** Xiaofang Wan, Maixian Liu, Mingze Ma, Danyang Chen, Na Wu, Li Li, Zhongjun Li, Guimiao Lin, Xiaomei Wang, Gaixia Xu

**Affiliations:** ^1^National-Regional Key Technology Engineering Laboratory for Medical Ultrasound, Guangdong Key Laboratory for Biomedical Measurements and Ultrasound Imaging, Department of Biomedical Engineering, School of Medicine, Shenzhen University, Shenzhen, China; ^2^Key Laboratory of Optoelectronics Devices and Systems of Ministry of Education/Guangdong Province, College of Optoelectronic Engineering, Shenzhen University, Shenzhen, China; ^3^Carson International Cancer Center, School of Medicine, Shenzhen University, Shenzhen, China; ^4^Department of Physiology, School of Basic Medical Sciences, Shenzhen University, Shenzhen, China; ^5^Collaborative Innovation Center for Optoelectronic Science and Technology, College of Optoelectronic Engineering, Shenzhen University, Shenzhen, China

**Keywords:** CuS@BSA, phototherma, *in vivo*, toxicity, HeLa cells

## Abstract

Nanomaterials with localized surface plasmon resonance (LSPR) have exquisite optical properties, which allow a wide range of applications. Non-stoichiometric copper sulfides with active LSPR have drawn great attention, because its LSPR peak falls in the NIR region that is suitable for deep bioimaging and photothermal therapy (PTT). Despite numerous biomedical applications, the biocompatibility and toxicity of copper sulfides have not been studied systematically. In this contribution, we synthesized the ultrasmall biocompatible copper sulfide nanoparticle encapsulated within bovine serum albumin (BSA), CuS@BSA. The physical features of CuS@BSA were characterized. The MTT and flow cytometry assays were performed. The *in vitro* PTT was also investigated. The results indicated that such CuS@BSA nanoparticle had an average TEM size of 8 nm, and an average DLS size of 15 nm. A lower concentration of CuS@BSA was not toxic to HeLa cells, but the critical apoptotic events occurred in HeLa cells after co-incubation with 45 μg/mL CuS@BSA for 48 h. The photothermal effect of the CuS@BSA in aqueous medium were concentration-dependent and time-dependent, which were also verified by flow cytometry and microscopy, while the CuS@BSA were co-cultured with HeLa cells and treated with laser. This work designed an ultrasmall potential biocompatible nanoparticle, CuS@BSA, for cancer photothermal therapy, and provided the toxic information to safely guide its biomedical applications.

## Introduction

With the rapid development of nanotechnology, many kinds of nanomaterials with unique physical and chemical properties have been designed, synthesized and applied in various fields (Liu and Swihart, 2014). Unique phenomena observed in nanomaterials (Dutta and Dolui, 2008), such as surface plasmon resonance (SPR) (Zhu and Su, 2013) and localized surface plasmon resonance (LSPR) (Choi et al., 2011), are particularly important optical properties that are increasingly playing important roles in biomedical and optoelectronic applications (Ozbay, 2006; Dreaden et al., 2012). In particular, the nanoparticles with high near-infrared absorption/emission are helpful for deep *in vivo* theranostics, such as photothermal therapy (PTT) and bioimaging (Dreaden et al., 2012). Generally, besides the surgery, radiotherapy and chemotherapy are most commonly accepted by clinical cancer therapy (Liu et al., 2013). However, it is well know that these methods could destroyed the tumor, as well as the normal cells and the immune system, which caused more serious consequences to patients (Kievit and Zhang, 2011). Fortunately, novel tumor therapeutic strategies, such as immunological therapy, biological therapy, PTT, and photodynamics therapy, have given hope to patients, because of the high selectivity, minimal invasion, and adverse effects (Choi et al., 2011; Zhang et al., 2015).

In recent years, more and more photothermal materials have been studied by researchers, such as Au, Ag and Pd-based novel metal nanoparticles. However, long-term safety remains a concern, which impedes them from being widely used in clinical applications (Nel et al., 2006; Liu et al., 2013). Thus, developing PTT agents that consist of naturally occurring substances in organisms would be highly beneficial for *in vivo* applications. Because such agents can effectively avoid serious adverse effects caused by the long-term retention of foreign substances in patients, and biodegradation through metabolism (Liu et al., 2013). Benefiting from the high optical extinction coefficient in the near-infrared region, various gold nanostructures, including gold nanoparticles (Geng et al., 2012), nanorods (Huang et al., 2010), nanostars (Yuan et al., 2012), nanocage (Yavuz et al., 2009), and nanoshells (Liu et al., 2011), have become the most popular photothermal agents in near-infrared PTT. Nevertheless, the high cost, long retention, and shape-dependent photothermal effects, limited their further applications. Some two dimensional materials, including Bi_2_Se_3_ (Song et al., 2016), WO_3_ (Balandeh et al., 2015), graphene (Chen et al., 2015), and black phosphorus (Jiang et al., 2016) have also been studied by researchers. Recently, the alternative agent of Au, copper sulfide nanoparticles (CuS NPs), have been investigated widely by researchers due to their outstanding absorbance, effective thermal conversion, and simple preparation (Goel et al., 2014; Liang et al., 2018). In particular, CuS NPs with strong NIR absorbance and photo stability, are ideal candidates for *in vivo* applications (Zhou et al., 2010). However, despite the positive results for *in vitro* and *in vivo* biomedical applications, we know little about the toxicity of CuS nanostructures. Zhang et al. (2015) found that the BSA-CuS nanoparticle had a cell viability of 90% at a concentration of 230 mg/L. Liang et al. (2018) performed a long-term toxicity study with a large dose of CuS@MSN and the results showed only minimal toxicity for living female BALB/c mice. Feng et al. (2015) studied the toxicity of CuS nanosheets for different types of cells. They found that nanosheets was non-toxic to HeLa, KB, and RAW 264.7 cells, while the nanosheets concentrations were below 50 μg/mL, and the cell viability decreased while the concentration was above 100 μg/mL. However, the more systematical toxicity study should be performed to advance the PTT application of CuS. A better understanding of the toxicity characteristics of CuS nanostructures will facilitate the development of a safe and effective PTT agent for cancer therapy (Liang et al., 2018).

In this work, we synthesized an ultrasmall copper sulfide nanoparticle encapsulated in BSA. The MTT assay and flow cytometry assay were performed to analyze the toxicity of such CuS@BSA nanoparticle. PTT was performed and considerable cell apoptosis was observed after irradiating the cells with a 1064 nm laser for 6 min. The flow cytometry and microscopy were used to investigate the PTT efficiency quantitatively and qualitatively, respectively. The results showed that the nanoparticle inhibited cell proliferation at a concentration of 45 μg/mL for 48 h co-incubation. The PTT was concentration-dependent and time-dependent. These results suggest that optimization of concentration and irradiation time are necessary for the safety of CuS@BSA nanoparticle in biomedical applications.

## Materials and Methods

### Chemicals

All chemicals and reagents used were analytical grade. Ultrapure water (Hangzhou Wahaha Group Co., Ltd., (Hangzhou, China) was used throughout this work. BSA was obtained from Yan cheng Sai bao Biotechnology Co., Ltd., (Jiangsu, China). CuCl_2_.2H_2_O and (NH_4_)_2_S were purchased from MACKLIN Co., Ltd., (Shanghai, China).

### Synthesis

The CuS@BSA nanoparticle was prepared by a simple method. Firstly, 100 mg of BSA was dissolved in 10 mL ultrapure water, and then the 250 μL CuCl_2_.2H_2_O solution at a concentration of 0.75 M was added to the above solution under magnetic stirring. Once the color of the mixture changed into blue, 100 μL of the (NH_4_)_2_S solution at a concentration of 3.2 M was added quickly. The mixture solution was subsequently heated up to 90°C for 30 min until the color turned to dark green, which indicated the formation of the CuS@BSA nanoparticle. The obtained CuS@BSA nanoparticle were stored at 4°C. The CuS@BSA nanoparticle used in the following experiments were dissolved in a PBS solution and filtered through a 220 nm film.

### Characterization

The morphology and microstructure of the CuS@BSA nanoparticle were characterized with a transmission electron microscope (TEM) (TecnaiG2 F20 S-TWIN, FEI, United States) operating at an accelerating voltage of 200 kV at room temperature. The samples for TEM were prepared by depositing a diluted nanoparticle solution into a 230-mesh Cu grid, sample droplets dried by water dispersion. The absorption spectra of CuS@BSA were measured by UV-vis (Lambda 750, PE, United States). The hydrodynamic diameters and size distribution of nanoparticles were characterized by dynamic light scattering technology on a Malvern Zetasizer (Zetasizer Nano ZS, Malvern, United Kingdom). CuS@BSA nanoparticle was diluted by ultrapure water before measurement.

### Colloidal Stability and Photothermal Performance

The stability of the BSA@CuS nanoparticle in the various media, such as pure water, PBS (pH = 7.4), FBS, and DMEM, was tested. The photothermal-induced temperature elevation was quantified by inserting a thermos-couple into the solution of nanoparticle irradiated with a 1064 nm laser. The solutions of the CuS@BSA nanoparticle with different concentrations (22.5, 45, 90, and 180 μg/mL) were irradiated with the laser at a power density of 0.73 W cm^-2^ for 3 min to evaluate the photothermal performance.

### Cell Cultures

The human cervical carcinoma cells, HeLa, were obtained from American Type Culture Collection (ATCC) and cultured in Dulbecco’s Modified Eagle’s Medium (DMEM, Gibco, United States), supplemented with 10% fetal bovine serum (FBS, Gibco, United States) and 100U penicillin/streptomycin (Gibco, United States). All cells were cultured at 37°C in a humidified atmosphere with 5% CO_2_.

### MTT Assay for BSA@CuS Nanoparticle

The cell viability of HeLa cells was evaluated through an MTT (Sigma-Aldrich, United States) assay. The cells were seeded into a 96-well plate (5 × 10^3^ cells/well) and incubated for 24 h at 37°C under 5% CO_2_. The cells were subsequently co-incubated with different concentrations of the CuS@BSA nanoparticle for 24 h or 48 h. The MTT solution (5 mg/mL) was added into cells for 10 μL/well. After 4 h of incubation, the culture medium was removed carefully and DMSO (150 μL/well) was added to sufficiently dissolve the blue crystals. The plates were assayed by a micro plate reader (Multiskan FC, Thermo Fisher, Finland) at a wavelength of 570 nm. The cell viability was calculated by normalizing the absorbance of the treated wells vs the control wells.

### Cell Apoptosis Detected by Flow Cytometry

The cell apoptosis was measured with the Annexin V-FITC Apoptosis Detection Kit (BD PharmingenR United States). On the day before assay, cells were planted onto 6-well plates (10 × 10^4^ cells/well) and incubated for 24 h at 37°C under 5% CO_2_. For apoptosis detection, the experimental groups were co-cultured with CuS@BSA nanoparticle for 24 or 48 h, while the blank groups were treated with PBS. All cells were dissociated with a trypsin solution (Gibco, United States) without EDTA and collected by centrifugation at 1000 rpm for 5 min. After washed with pre-cooled PBS, cells were resuspended in 100 μL 1 × binding buffer. A 5 microliter Annexin V-FITC solution was added into cells and cells were incubated for 5 min at room temperature and protected from light. Finally, 10 μL propidium iodide (PI) solution and 400 μL PBS was added into cells. The signals of FITC fluorescence were detected by a flow cytometer (FACSAria II, BD, United States).

### *In vitro* Photothermal Therapy of CuS@BSA Nanoparticle

On the day before assay, cells were planted onto 6-well plates (10^4^ cells/well) and incubated for 24 h at 37°C under 5% CO_2_. HeLa cells were treated without or with the CuS@BSA nanoparticle, and exposed to the 1064 nm laser with a power density of 0.55 W cm^-2^ for 0–5 min. The dyes, Calcein-AM and PI, were used to stain the population of viable and necrotic cells, respectively. The Calcein-AM was incubated with cells for 30 min at 37°C. Later, the cells were washed three times with PBS to remove the Calcein-AM dye. Finally, 10 μL PI was added into cells samples at a final volume of 100 μL.

### Data Statistics

MTT experimental data were expressed as mean ± standard deviation (SD). Multigroup comparisons of the means were carried out through a one-way analysis of a variance (ANOVA) test.

## Results and Discussion

### Characterization of BSA–CuS Nanoparticle

**FIGURE 1:**
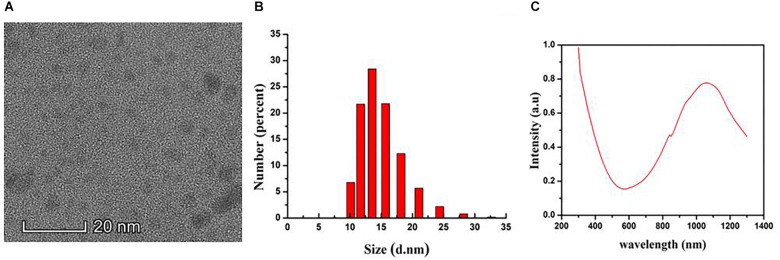
The Characterization of the CuS@BSA nanoparticle. **(A)** A TEM image. Scale bar: 20 nm. **(B)** DLS analysis. **(C)** Absorbance spectrum.

The morphology of the CuS@BSA nanoparticle were shown as a TEM image (Figure 1A). It demonstrated a relatively monodispersed size distribution with an averaged size of 8 nm. The hydrodynamic diameters of the aqueous nanoparticle were 15 nm (Figure 1B). The NIR absorption of the CuS@BSA nanoparticle were measured by UV-vis, and the absorption peak was at around 1060 nm (Figure 1C). The molar extinction coefficient is 7.06 × 10^9^ cm^-1^ M^-1^.

### Cytotoxicity of CuS@BSA Nanoparticle

**FIGURE 2:**
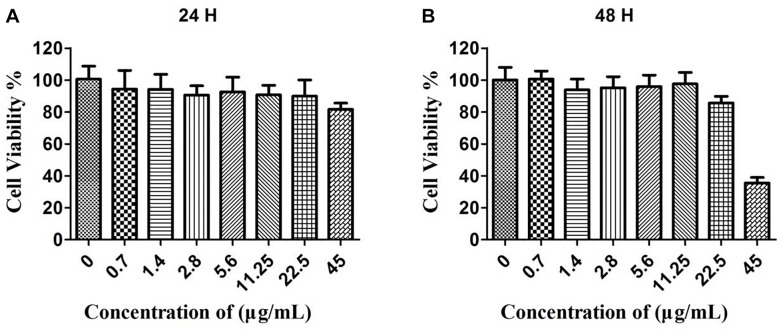
The cytotoxicity of CuS@BSA nanoparticle for HeLa cells. **(A)** The HeLa cells were co-cultured with the CuS@BSA nanoparticle for 24 h. **(B)** The HeLa cells were co-cultured with the CuS@BSA nanoparticle for 48 h. *n* = 6.

In order to evaluate the cytotoxicity of the CuS@BSA nanoparticle, HeLa cells were co-incubated with a CuS@BSA nanoparticle at different concentrations for 24 h and 48 h. The standard MTT assay was carried out to determine the cell viability. For the case of HeLa cells treated for 24 h, the cell viability remained above 90% when the applied CuS@BSA nanoparticle concentrations ranged from 0.7 to 22.5 μg/mL for 24 and 48 h. It was worth noting that there was a special phenomenon once the cells were treated with the CuS@BSA nanoparticle at the concentration of 45 μg/mL. When the treatment time was 24 h, the cell viability remained above 80% (Figure 2A). When the treatment time was prolonged to 48 h, the cell viability was only about 35% (Figure 2B), which indicated that the CuS@BSA nanoparticle was a concentration- and time-dependent cytotoxicity pattern in the cell viability.

### Cell Apoptosis Assay After Co-cultured With CuS@BSA Nanoparticle

**FIGURE 3:**
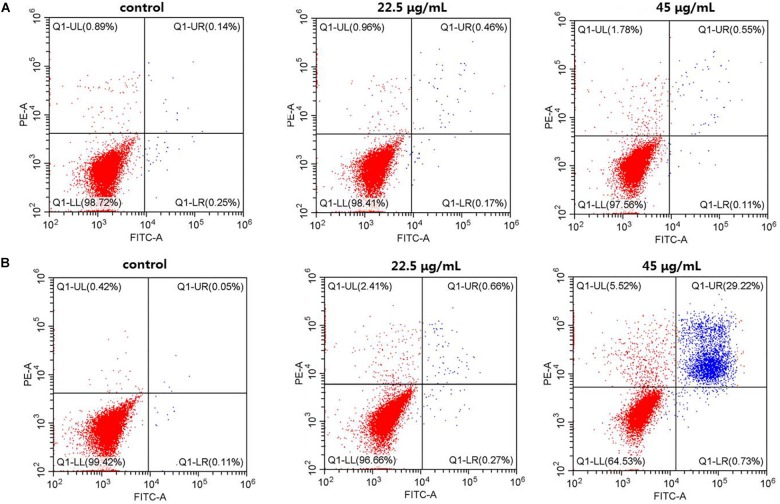
CuS@BSA nanoparticle infected HeLa cells analyzed by Flow cytometer, stained with annexin V-FITC/PI. **(A)** The HeLa cells were co-cultured with the CuS@BSA nanoparticle for 24 h; **(B)** The HeLa cells were co-cultured with the CuS@BSA nanoparticle for 48 h.

To systematically assess the toxicity of the CuS@BSA nanoparticle on cells, the fraction of apoptotic cells caused by the nanoparticle were analyzed by flow cytometry (Figure 3). Here, only the cells treated with nanoparticles at a higher concentration were investigated. For the case of HeLa cells treated with the CuS@BSA nanoparticle at a concentration of 22.5 and 45 μg/mL for 24 h, the cell apoptosis rates of the CuS@BSA nanoparticle in all groups were comparable to that in the control groups. When the co-incubation time was extended to 48 h, the cell apoptosis rates showed no obvious change, while the HeLa cells were treated with the CuS@BSA nanoparticle at the concentration of 22.5 μg/mL. However, when the concentration of the CuS@BSA nanoparticle was increased to 45 μg/mL, the cell apoptosis rates increased dramatically to 29.22%.

### The Photothermal Performance

**FIGURE 4:**
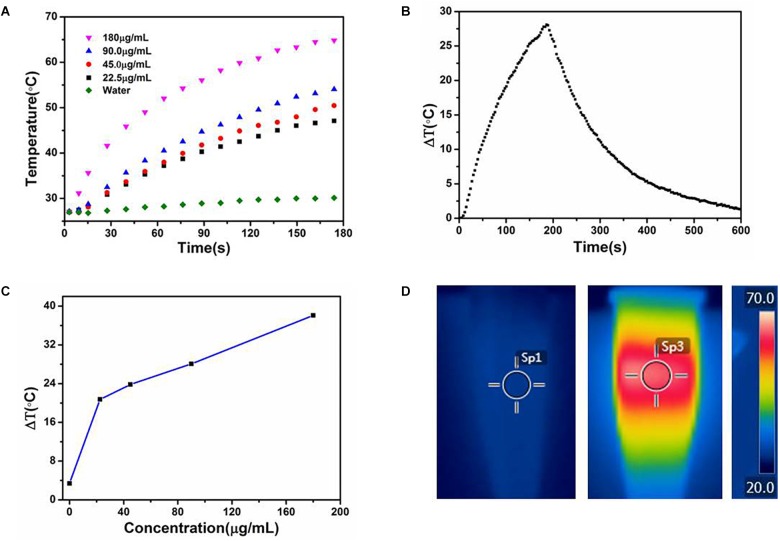
The photothermal performance of the CuS@BSA nanoparticle. **(A)** Temperature changes of water and CuS@BSA nanoparticle aqueous solution at different concentrations with irradiation time. **(B)** The temperature changes of the CuS@BSA nanoparticle aqueous solution (90 μg/mL) for 180 s with NIR laser (1064 nm, 0.73 W/cm^2^) and then the laser was shut off. **(C)** Plot of temperature change (ΔT) over a period of 180 s vs. the concentration of CuS@BSA nanoparticle. **(D)** The infrared images of CuS@BSA nanoparticle aqueous solution. Left and middle images were the infrared images of CuS@BSA nanoparticle aqueous solution in tube before and after being irradiated by NIR laser, respectively. The right image was the scale bar of temperature. The color represented various temperature. Sp1 and Sp3 were marked as the points that the temperature were recorded.

The strong NIR absorption of the CuS@BSA nanoparticle motivated us to evaluate their photothermal conversion ability. The quantitative temperature changes of the CuS@BSA in an aqueous solution at different concentrations with time were measured. CuS@BSA nanoparticle was dispersed in water at concentrations ranging from 22.5 to 180 μg/mL, and then irradiated with a 1064 nm laser (a continuous wave fiber-coupled laser, 0.73 W/cm^2^) for 180 s. Pure water was used as a control. As shown in Figure 4A, the temperatures of all the CuS@BSA nanoparticle samples increased with the irradiation time, and the temperature increased more rapidly when increasing the concentration of CuS@BSA nanoparticle. The photothermal conversion efficiency of CuS@BSA nanoparticles used in this work was 69.37%. After irradiation for 180 s, the temperature of the CuS@BSA nanoparticle aqueous solution was increased by 40°C at a concentration of 180 μg/mL (Figure 4C).

### *In vitro* Photothermal Efficiency of CuS@BSA Nanoparticle

**FIGURE 5:**
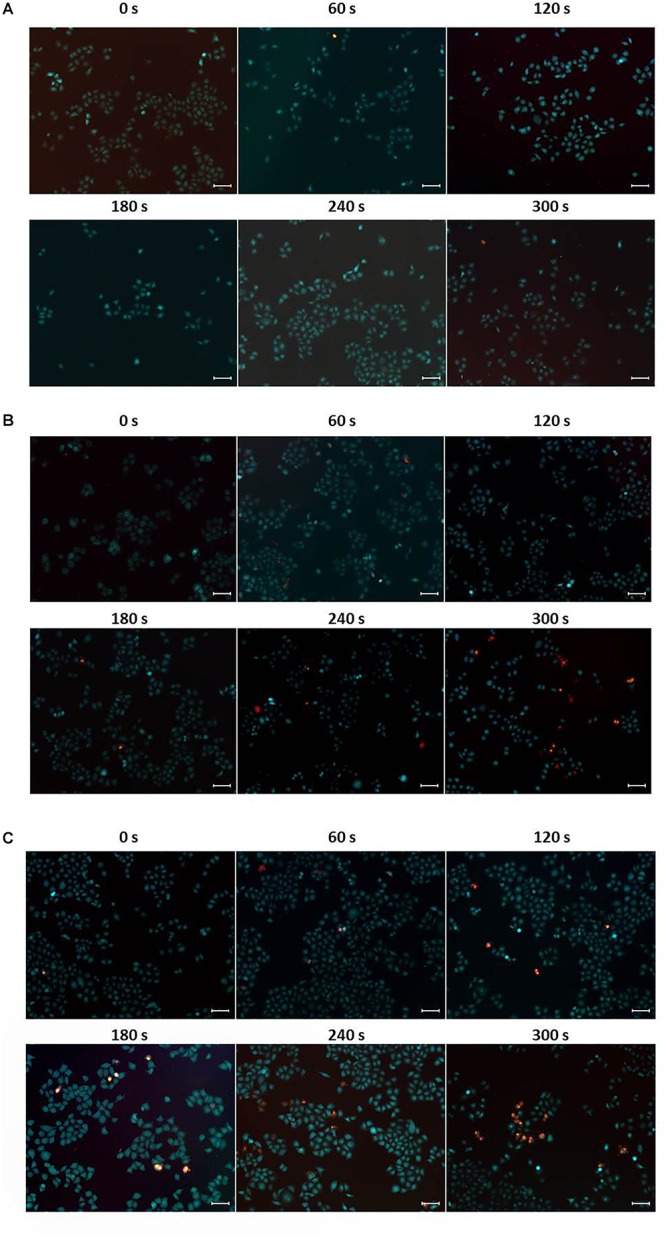
Typical fluorescence microscopic images of HeLa cells, stained with calcein AM (green, live cells) and PI (red, dead cells), respectively. **(A)** HeLa cells without CuS@BSA nanoparticle treatment. **(B)** HeLa cells treated with CuS@BSA nanoparticle at a concentration of 22.5 μg/mL. **(C)** HeLa cells treated with CuS@BSA nanoparticle at a concentration of 45 μg/mL. All the HeLa cells were irradiated by the 1064 nm laser with a power density of 0.55 W cm^-2^ for 0–5 min. Scale bar:100 μm.

The high photothermal conversion efficiency and low cytotoxicity of CuS@BSA nanoparticle prompted us to evaluate their feasibility as a PTT agent for cancer therapy. The HeLa cells co-cultured with the CuS@BSA nanoparticle were irradiated by 1064 nm laser. The inverted luminescence microscope was used to distinguish the live cells and dead cells qualitatively. The flow cytometry analysis was applied to calculate quantitatively the cell viability. According to the *in vitro* toxicity results, we chose two concentrations, 22.5 and 45 μg/mL, for PTT cancer cell treatment. The microscopic images showed that the irradiating laser had no obvious destructive effects on the HeLa cells without CuS@BSA nanoparticle treatment, even after being irradiated for 360 s (Figure 5A). However, the live cells decreased and the dead cells increased over the irradiation time while the HeLa cells were treated with CuS@BSA nanoparticles (Figure 5B,C). In comparing the fluorescence images of cells treated with CuS@BSA at different concentration levels but for the same amount of irradiation time, no obvious differences were observed.

**FIGURE 6:**
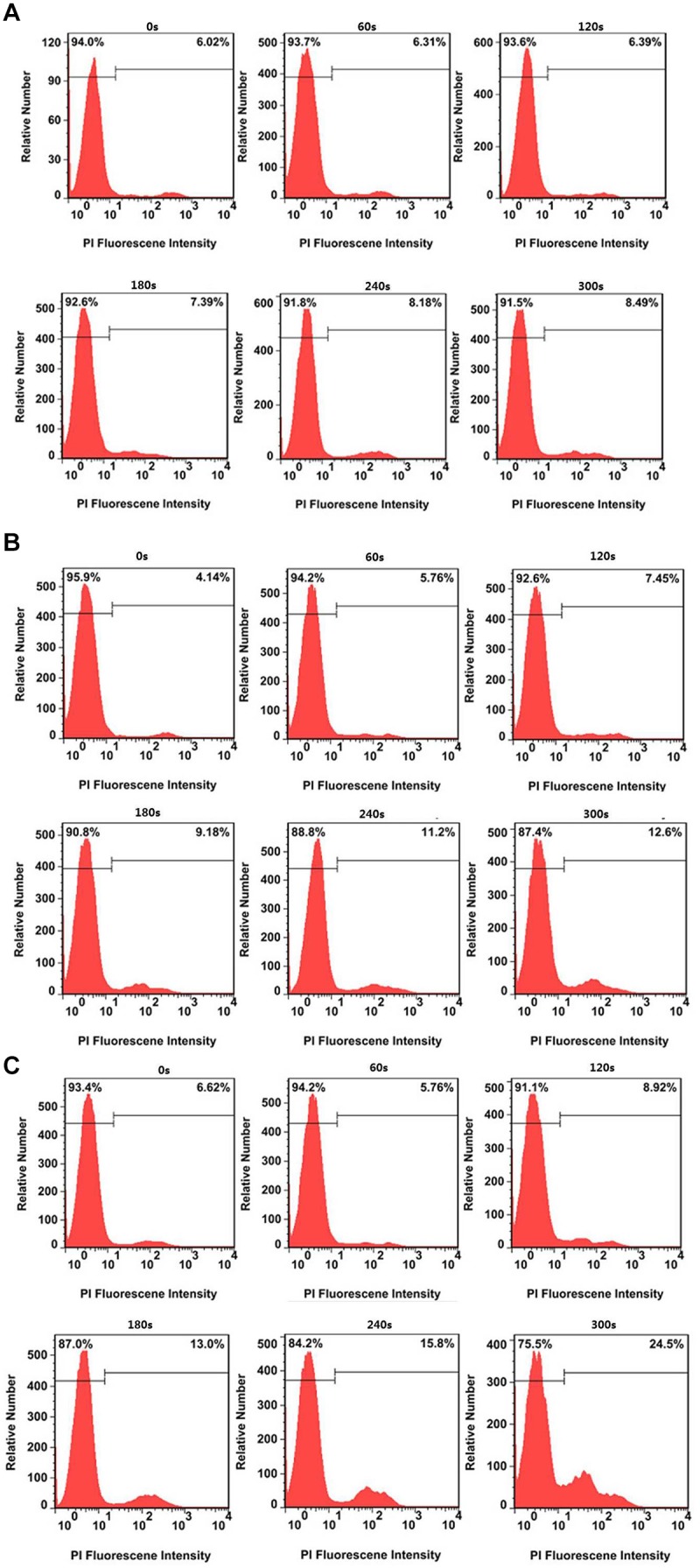
Typical cell viability after treatment without and with CuS@BSA nanoparticles over irradiation time. **(A)** HeLa cells treated with different laser irradiation time without CuS@BSA nanoparticles. **(B)** HeLa cells treated with the CuS@BSA nanoparticle at a concentration of 22.5 μg/mL. **(C)** HeLa cells treated with the CuS@BSA nanoparticle at a concentration of 45 μg/mL. All the HeLa cells were irradiated by the 1064 nm laser with a power density of 0.55 W cm^-2^ for 0–5 min.

The flow cytometry analysis provides the quantitative photothermal efficiency of CuS@BSA nanoparticle for *in vitro* cell PTT. The results showed that the dead cells increased slightly, from 6.02 to 8.49%, while the HeLa cells were treated without nanoparticles over the irradiation time (Figure 6A). When the HeLa cells were treated with the CuS@BSA nanoparticle at a concentration of 22.5 μg/mL, the dead cell percentage increased significantly, from 4.14 to 12.6% (Figure 6B). When the HeLa cells were treated with the CuS@BSA nanoparticle at a concentration of 45 μg/mL, the dead cell percentage increased dramatically, from 6.62 to 24.5% (Figure 6C).

## Discussion

In recent years, many methods for synthesizing the CuS nanoparticle have been developed, and the toxicity of CuS nanoparticle has also been studied. However, the synthetic methods currently known are complicated, and the toxicity study on CuS nanomaterial is not clear enough. Therefore, this study used a protein BSA as a coating material to synthesize the CuS nanoparticle in a fast and simple way, and systematically studied the toxicity and its photothermal performance of this nanoparticle.

It is well known that BSA is an excellent biomolecule for nanoparticle surface modification due to its large quantity, low cost, and good biocompatibility. The BSA contains 35 potential thiol groups, including 17 disulfide bonds and 1 free cysteine, which can be used to stabilize the affinity of CuS nanoparticle due to the strong binding between Cu and thiol groups (Zhang et al., 2015). Here we synthesized ultrasmall CuS@BSA nanoparticle in a fast and simple way. The TEM size was only around 8 nm, which was suitable for further functionalization and applied in *in vitro*/*in vivo* biosensing, bioimaging and therapy. The NIR absorption of the CuS@BSA nanoparticle was at 1060, which was in the range of the biological tissue optical window nm and suitable for *in vivo* PTT applications.

The safety assessment of the nanoparticle is the most important thing before we started the biomedical application. In this work, the MTT and flow cytometry assays were performed to investigate the cytotoxicity and apoptosis of CuS@BSA nanoparticles. The results indicated that the toxicity of such nanoparticle was time- and concentration-dependent. The longer the co-incubation time and the higher the concentrations that were applied, the more cells were apoptosis-induced. There are two speculations to explain the cell apoptosis. One consideration was that that too much nanoparticle entered the cells and resulted in cells dysfunction (Guo et al., 2018), which further caused apoptosis. The other one was that the Cu^2+^ leakage induced apoptosis (Nordin et al., 2018). Further work should be carried out to investigate the appropriate pathway. The toxicity assessment could only provide a safe concentration range for biomedical application.

Generally, the photothermal conversion was nanoparticle concentration-dependent (Habash et al., 2007; Feng et al., 2015; Zhang et al., 2015). According to the cytotoxicity assay, CuS@BSA at the concentration of 22.5 μg/mL was the highest safe concentration for an *in vitro* cells study. In this work, the photothermal performance of the CuS@BSA nanoparticle in an aqueous solution at a concentration from 22.5 to 180 μg/mL was performed, in order to further choose the appropriate concentration range of nanoparticles under the safe premise. To our knowledge, it was the highest photothermal conversion efficiency of the CuS nanoparticle as a PTT sensitive agent at a similar CuS nanoparticle concentration (Feng et al., 2015; Zhang et al., 2015; Yang et al., 2016; Liang et al., 2018). In contrast with other reports, our results showed that the temperature would increase rapidly after treatment with minutes-irradiation. Even at the lowest concentration, 22.5 μg/mL, the temperature increase by 26°C after 180 s irradiation time. It has been reported that cancer cells can be killed after maintenance at 42°C for 15–60 min (Habash et al., 2007). The duration can be shortened to 4–6 min for temperatures over 50°C. Thus, our CuS@BSA nanoparticle would reduce the irradiation time and has great potential as an excellent PTT agent candidate.

The *in vitro* PTT experiments verified the excellent photothermal conversion performance of the CuS@BSA. The PTT efficiency of the nanoparticles for HeLa cells was obvious irradiation time-dependent. In addition, over the same irradiation time, with the concentration increasing, the dead cell percentage also increased. The dead cell increased from 12.6 to 24.5% when the concentration of CuS@BSA increase from 22.5 to 45 μg/mL. However, in order to obtain the better therapy efficiency, we should keep the balance between the cytotoxicity and the photothermal conversion and choose the appropriate concentration of the nanoparticles.

## Conclusion

In summary, we have reported a novel strategy to synthesize the biocompatible CuS@BSA nanoparticle for *in vitro* PTT application. The characterization results showed that the size of the CuS@BSA nanoparticle particle was 8 nm, which was suitable for both *in vitro* and *in vivo* biomedical applications. The CuS@BSA was not toxic for HeLa cells at a concentration lower than 22.5 μg/mL for 48 h, but the critical apoptotic events occurred in HeLa cells after co-incubation with 45 μg/mL CuS@BSA for 48 h. The photothermal experiments demonstrate that our CuS@BSA nanoparticle is characterized with a high photothermal conversion, a short irradiation time and a long recovery time. The photothermal effect of the CuS@BSA in an aqueous medium was concentration-dependent and time-dependent. Thus, this work provided a fast and simple way to synthesize an ultrasmall biocompatible nanoparticle, CuS@BSA, which had an excellent photothermal conversion feature, and could be an excellent PTT agent candidate in future clinics.

## Author Contributions

XW designed the experiments, conducted the basic work, and prepared the initial draft. ML designed the method to synthesize the CuS@BSA nanoparticles. MM performed the microscopic imaging. DC assisted in preparing the cells. NW conducted the photothermal experiments. LL analyzed the MTT data and proposed the data interpretation. ZL provided assistance for laser using and parameters choosing. GL helped to analyzed the apoptosis data. XW provided the statistical analysis and edited the manuscript. GX supervised the work and revised the manuscript.

## Conflict of Interest Statement

The authors declare that the research was conducted in the absence of any commercial or financial relationships that could be construed as a potential conflict of interest.
